# Retrospective observational cohort study on innovation in oncology and progress in survival: How far have we gotten in the two decades of treating patients with advanced non-small cell lung cancer as a single population?

**DOI:** 10.1371/journal.pone.0232669

**Published:** 2020-05-12

**Authors:** Nahila Justo, Jonas Nilsson, Beata Korytowsky, Johan Dalen, Terri Madison, Alistair McGuire

**Affiliations:** 1 Department of Neurobiology, Care Sciences and Society, Karolinska Institute, Solna, Sweden; 2 ICON Plc, ICO, Stockholm, Sweden; 3 United States Health Economics Outcomes Research, Bristol-Myers Squibb, Lawrenceville, NJ, United States of America; 4 ICON Plc, ICO, Lexington, KY, United States of America; 5 Department of Health Policy, London School of Economics, London, United Kingdom; West China hospital, Sichuan University, CHINA

## Abstract

We assessed the impact of new antineoplastic agents on the overall survival (OS) of advanced non-small cell lung cancer (aNSCLC) patients followed up until 2012. Multivariate regression models were run for OS (outcome) and four proxies for innovation (exposure): Index (InnovInd, for SEER-Research data 1973–2012) and three levels of aggregation of Mean Medication Vintage, i.e. Overall (MMV_Overall_), using data aggregated at the State Level (MMV_State_), and using patient-level data (MMV_Patient_) using data from the US captured in SEER-Medicare 1991–2012. We derived Hazard ratios (HR) from Royston-Parmar models and odds ratios (OR) from a logistic regression on 1-year OS. Including 164,704 patients (median age 72 years, 56.8% stage IV, 61.8% with no comorbidities, 37.8% with adenocarcinoma, 22.9% with squamous-cell, 6.1% were censored). One-year OS improved from 0.22 in 1973 to 0.39 in 2012, in correlation with InnovInd (r = 0.97). Ten new NSCLC drugs were approved and 28 more used off-label. Regression-models results indicate that therapeutic innovation only marginally reduced the risk of dying (HROverall = 0.98 [0.98–0.98], HR_MMV-Patient_ = 0.98 [0.97–0.98], and HR_MMV-State_ = 0.98 [0.98–0.98], and slightly improved 1-year survival (OR_MMV-Overall_ = 1.05 95%CI [1.04–1.05]). These results were validated with data from the Swedish National Health Data registers. Until 2013, aNSCLC patients were treated undifferentiated and the introduction of innovative therapies had statistically significant, albeit modest, effects on survival. Most treatments used off-guidelines highlight the high unmet need; however new advancements in treatment may further improve survival.

## Introduction

Worldwide, lung cancer remains the most commonly occurring malignant neoplasm with 1.8 million new cases in 2012 (12.9% of all new cancer cases), and the most common cause of death from cancer accounting for 1.6 million lives lost (19.4% of all cancer-related deaths)[1]. In the United States (US), 218,527 new cases were diagnosed in 2015 and 153,718 deaths were registered. The majority of lung cancers are non-small cell lung cancer (NSCLC) and diagnosed when inoperable locally advanced (Stage IIIB) or metastatic (Stage IV)[2–5]. While 5-year survival rates for the overall lung cancer patient population improved almost 60% between 1975–1977 and 2008–2014, those diagnosed with advanced or metastatic NSCLC (aNSCLC) still carry very poor prognosis. In the 1970s, the median overall survival for patients with aNSCLC was six months; and by 2012, it had barely surpassed nine months[6].

Historically, treatment options have been limited[6] and consisted of successive generations of chemotherapy (anthracyclines, alkylating agents like platinum-based compounds, and taxanes) that did not differentiate patients by histology, tumor profile or specific biomarkers[7]. While Lichtenberg and colleagues have proven that pharmaceutical innovation has positively affected the life expectancy of cancer patients in general[8, 9], the limited effectiveness in aNSCLC warrants additional research.

Therefore, we conducted a thorough account of the level of therapeutic innovation introduced between 1991 and 2012 in the treatment of patients diagnosed with aNSCLC and an analysis of its impact on survival.

## Materials and methods

This was a retrospective observational cohort study on patients diagnosed with aNSCLC between 1991 and 2012, in the US, selected according to the following criteria: a primary diagnosis of advanced or metastatic NSCLC microscopically-confirmed. Patients were excluded if they met any of the following criteria: diagnosed at autopsy or within 30 days of death date, neuroendocrine tumours, younger than 18, or disease stage earlier than IIIA as defined by the American Joint Committee on Cancer (AJCC) classification.

We extracted patient-level data from the linked database SEER-Medicare (Carrier Claims, Outpatient Claims and Medicare Provider Analysis and Review and Prescription Drug Event File)[4]. In order to assess a longer-term trends in survival, we also analyzed two extended cohorts of patients diagnosed between 1973 and 2012, with data extracted from the SEER Research database and from the Swedish National Health Data registers (Cancer Register, Cause of Death Register and Patient Register)[10]. Though no patient-level treatment data was available for those additional 18 years in either country so only aggregated analyses were performed.

Additionally, we conducted a targeted literature review in MEDLINE and EMBASE to gather necessary information on oncology therapies introduced during these years. The review was complemented with targeted searches in the archives of the US Food and Drug Administration (FDA)[11], the European Medicines Agency (EMA)[12], the Swedish Medicinal Agency (Läkemedelsverket)[13], and the Clinical Outcome Labelling Claims Database (PROLabels^™^). These searches aimed at identifying marketing authorizations for treatments approved in aNSCLC with the respective dates, as well as evidence and date of first use for treatments without a labelled aNSCLC indication (i.e., off-label).

All methods were carried out in accordance with relevant guidelines and regulations and the protocols were approved by the respective institutional and/or licensing committee. In the US, this study was approved by Quorum Review IRB on May 20, 2015 with registration number 30556/1. In Sweden, this study was approved by the Regionala Etikprövningsnämnden in Stockholm on March 25, 2015 with registration number 2015/406-31/4. Following the norms from these authorities, no informed consent from the subjects was required.

### Definition of innovation

Following on Lichtenberg’s work, this study defines innovation in oncological treatments in relation to their year of introduction[14, 15], and builds four proxies. The first proxy is the Innovation Index (InnovInd), defined as the accumulated sum of aNSCLC systemic treatments available by year of approval, or evidence of earlier off-label use if confirmed in the literature. This proxy was assembled for the US and for Sweden for the period 1973–2012, and used to evaluate the long-term trend analysis of aggregated data.

The other three proxies incorporate actual usage to account for speed of uptake of new therapies. Due to the availability of patient-level treatment data, they only cover the period 1991–2012 in the US. These were defined by the Mean Medication Vintage (MMV) of aNSCLC systemic treatments used per year (i.e., year of introduction weighted by the share of patients using that given treatment in each year, regardless of treatment line). Further, three levels of aggregation were used for the MMV: a) MMV_overall_: estimated for each cohort defined by the year of diagnosis, b) MMV_state_: clustered geographically by state in each year, and c) MMV_patient_: individual-patient level estimates per year.

### Statistical analyses

We present descriptive statistics and graphical illustrations of the historical trajectory of exposure, outcomes and main covariates. We measured the correlation between innovation (InnovInd) and overall survival (OS) with the Pearson's correlation coefficient (r).

We conducted a Cox proportional-hazard (CPH) regression, with survival time in days as the outcome and innovation in aNSCLC treatments as the exposure. Covariates included in the models were gender, age of diagnosis, Charlson Comorbidity Index (CCI) (16), histology, residence and race (US only). Patients who were still alive or lost to follow-up at the end of the study period were censored. We tested the proportional hazard assumption using Schoenfeld residuals. If the assumption of proportionality was not fulfilled, to relax the assumption of linearity of log time, we built a Royston-Parmar flexible model [16] by using restricted cubic splines for those variables at risk. In both cases, we estimated hazard ratios (HR) with 95% CIs and p-values.

Additionally, we ran a logistic regression using one-year survival as a dichotomous dependent variable with the same exposure proxies and covariates as in the previous models and estimated odds ratios with 95% CIs and p-values.

## Results

**Fig 1:**
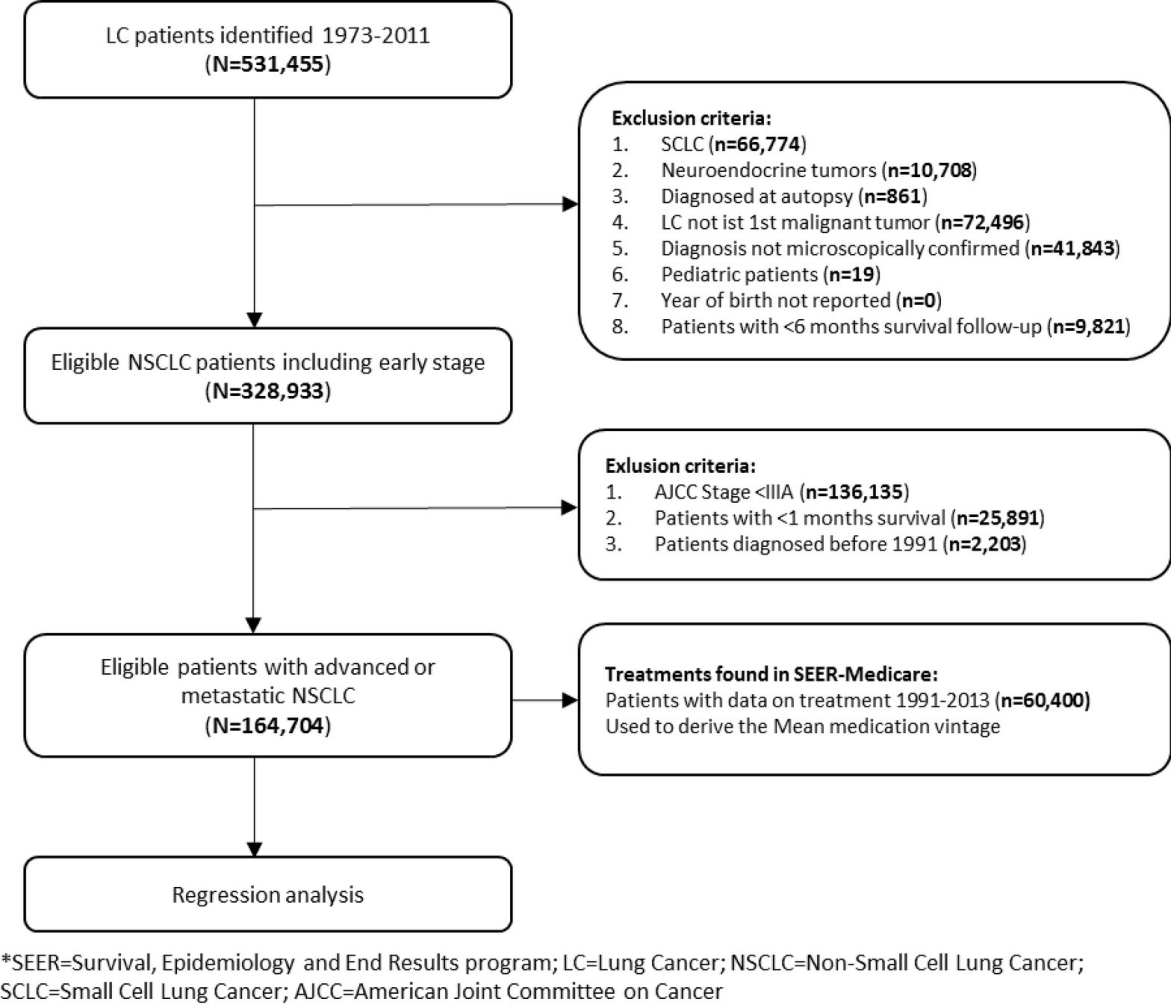
Patient selection flow chart, SEER Medicare.

We extracted the records of 164,704 patients diagnosed between 1991 and 2012 who met the inclusion and exclusion criteria, of which 60,400 received at least one line of active treatment. The majority of patients were excluded because NSCLC was a secondary tumor, or they presented with different histologies (small-cell and neuroendocrine) (Fig 1). The 10,076 (6.12%) patients who were still alive at the end of follow-up, plus 201 (0.12%) lost to follow-up, were subject to censoring.

Table 1 presents the main characteristics of the patient population under analysis (SEER Medicare 1991–2012). The median age was 72 years, ranging between 21 and 104 years old. The majority of patients were men (56.8%) with metastatic disease (stage IV accounts for 56.8%) and of white race (79.9%). The most common histology was adenocarcinoma, followed by squamous-cell carcinoma; however, 24.1% of patients had an unconfirmed histology. Table 1 also depicts a baseline comparison of patient characteristics with a cohort from the more representative SEER Research database, selected using the same criteria presented in Fig 1. The main difference is that the population under study is older than overall aNSCLC population in the US.

**Table 1:** Main characteristics of the patient population and assessment of generalizability.

Patients’ characteristics	SEER-Medicare (N = 164,704)		SEER Research (N = 133,077)	
**Gender (male)**	93,514	56.8%	76,678	57.6%
**Age at diagnosis,** mean (SE)	71.5 (0.022)	NA	67.0 (0.03)	NA
**Age at diagnosis,** median (max; min)	72 (21; 104)	NA	68 (18; 104)	NA
**State of residence**				
California	58,949	35.8%	19,565	14.7%
Connecticut	11,147	6.8%	21,248	16.0%
Georgia	16,006	9.7%	11,508	8.6%
Hawaii	3,428	2.1%	6,248	4.7%
Iowa	10,080	6.1%	17,350	13.0%
Michigan	13,979	8.5%	25,917	19.5%
New Mexico	3,337	2.0%	6,755	5.1%
Utah	2,033	1.2%	4,082	3.1%
Washington	11,061	6.7%	20,404	15.3%
Kentucky	10,841	6.6%	NA	NA
Louisiana	8,912	5.4%	NA	NA
New Jersey	14,931	9.1%	NA	NA
**Race**				
White	131,590	79.9%	105,747	79.5%
Black	18,458	11.2%	16,374	12.3%
Asian	8,680	5.3%	7,043	5.3%
Other	5,976	3.6%	3,891	3.0%
**Histology**				
Adenocarcinoma	62,182	37.8%	50,083	37.6%
Squamous	37,707	22.9%	29,227	22.0%
Malignant carcinomas (NOS)	12,604	7.7%	14,428	10.8%
NSCLC	27,017	16.4%	16,081	12.1%
Large cell carcinoma	8,288	5.0%	8,055	6.1%
Other	16,906	10.3%	15,203	11.4%
**AJCC Stage (7**^**th**^ **edition)**				
IIIA	25,488	15.5%	14,613	11.0%
IIIB	45,722	27.8%	28,386	21.3%
IV	93,494	56.8%	65,650	49.3%
Not staged	0	0.0%	24,428	18.4%
**Charlson Comorbidity Index,** mean (SE)	0.71 (0.03)	NA	NA	NA

SEER = Surveillance, Epidemiology, and End Results, NOS = Not Otherwise Specified; SE = Standard Error; NA = Not Available or Not Applicable; NSCLC = Non-Small Cell Lung Cancer; AJCC = American Joint Committee on Cancer

**Fig 2:**
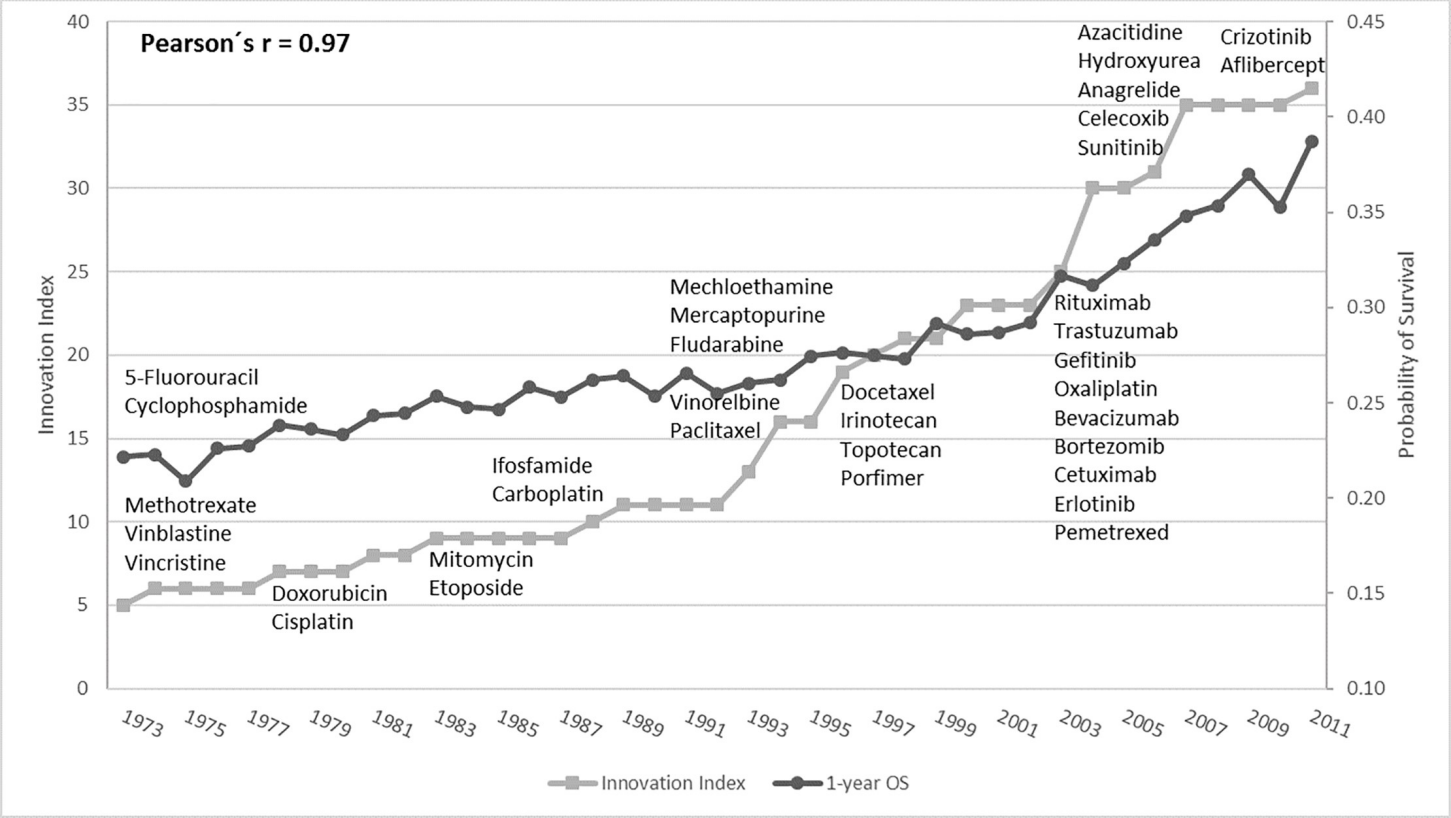
Innovation index and 1-year overall survival over time in the USA.

Between 1973 and 2012, median OS increased from five to nine months and 1-year OS rose from 0.22 to 0.38. Fig 2 reveals that the evolution of 1-year OS is highly correlated with the progressive introduction of new aNSCLC systemic therapies (r = 0.97); particularly after 1992, when most of the innovative treatments were introduced into the treatment armamentarium (Table 2). Between 1991 and 2012, we identified 38 therapies in use for aNSCLC, of which 28 (74%) did not have a labeled indication for aNSCLC. The other 10 therapies had received FDA approval for aNSCLC, many as part of label expansion. Notably, five of them were already in use for aNSCLC patients preceding their official indication. Table 2 presents a detailed account of the year and circumstances of introduction of each drug.

**Table 2:** Data used in the construction of the mean medication vintage proxy variables.

Active substance	FDA approval (primary indication)	FDA approval (in NSCLC)	Date of first use in Medicare	Number of treatments found in Medicare 1991–2013	First use for NSCLC	Type of evidence
5-Fluorouracil	1962		1991-01-16	1,474	1973	Off-label
Cyclophosphamide	1959		1991-01-10	516	1973	Off-label
Methotrexat	1953	Not mentioned	1991-01-03	2,015	1973	Off-label
Vinblastine Sulfate	1965		1991-01-24	1,098	1973	Off-label
Vincristine	1963		1991-03-08	349	1973	Off-label
Doxorubicin hydrochloride	1974		1991-02-20	466	1974	Off-label
Cisplatin	1978		1991-02-04	8,556	1978	Off-label
Mitomycin	1981		1991-02-07	1,065	1981	Off-label
Etoposide phosphate	1983		1991-02-22	6,251	1983	Off-label
Ifosfamide	1988		1991-03-19	160	1988	Off-label
Carboplatin	1989	no year (NCI)	1991-01-24	51,680	1989	Off-label
Mechlorethamine Hydrochloride	1949	no year (NCI)	1993-03-01	33	1993	Off-label
Mercaptopurine	1953		2007-01-03	34	1993	Off-label
Fludarabine	1991		1994-03-14	40	1994	Off-label
Paclitaxel	1992	1998-06-30	1994-01-03	35,056	1994	Earlier off-label
Vinorelbine tartrate	1994	1994-12-23	1995-03-03	11,317	1994	FDA approval
Docetaxel	1996	1999-12-23	1996-11-22	16,331	1996	Earlier off-label
Irinotecan Hydrochloride	1996		1998-04-10	937	1996	Off-label
Topotecan hydrochloride	1996		1998-01-08	348	1996	Off-label
Gemcitabine	1996	1998-08-25	1997-03-21	20,891	1997	Earlier off-label
Porfimer sodium	1995	1998-01-09	1998-04-14	20	1998	FDA approval
Rituximab	1997		2000-03-01	137	2000	Off-label
Trastuzumab	1998		2000-01-12	50	2000	Off-label
Gefitinib	2003	2003-05-05	2006-09-27	73	2003	FDA approval
Oxaliplatin	2002		2003-08-11	84	2003	Off-label
Bevacizumab	2004	2006-10-11	2004-09-07	8,399	2004	Earlier off-label
Bortezomib	2003		2004-07-26	21	2004	Off-label
Cetuximab	2004		2004-07-29	452	2004	Off-label
Erlotinib	2004	2004-11-18	2007-01-01	9,502	2004	FDA approval
Pemetrexed disodium	2004	2004-08-19	2004-05-18	13,437	2004	Earlier off-label
Azacitidine	2004		2006-02-01	22	2006	Off-label
Anagrelide	1997		2007-04-06	17	2007	Off-label
Celecoxib	1998		2007-01-01	3,257	2007	Off-label
Hydroxyurea	1967		2007-01-02	109	2007	Off-label
Sunitinib Malate	2006		2007-07-03	17	2007	Off-label
Crizotinib	2011	2011-08-26	2011-09-08	78	2011	FDA approval
Ziv-Aflibercept	2012		2012-07-06	17	2012	Off-label

FDA = Food and Drug Administration, NSCLC = Non-Small Cell Lung Cancer

The regression analyses produced consistent results with all three definitions of innovation, as can been in Table 3. The HR for the exposure MMV was 0.98 (95%CI 0.98–0.98 and 0.97–0.98 in the MMV_patient_ regression), indicating that newer, mostly targeted, treatments have had a beneficial, albeit small, influence on the survival of aNSCLC patients. The logistic regression of 1-year OS with an OR = 1.05 (95%CI 1.04–1.05) confirms these findings as do the analyses in Sweden with InnovInd 1991–2013 as exposure (Royston-Parmar flexible model with HR = 0.95 (95% CI 0.94–0.95) and logistic regression model with OR = 1.10 (95% CI 1.09–1.10)).

**Table 3:** Regression models with alternative definitions of innovation and survival.

Covariates	R-PFM MMV (overall)				R-PFM MMV (patient level)				R-PFM MMV (clustered per state)				Logistic regression MMV (overall)			
	(N = 164,704)				(N = 60,125)				(N = 164,704)				(N = 164,704)			
	HR	P>|z|	95% CI		HR	P>|z|	95% CI		HR	P>|z|	95% CI		OR	P>|z|	95% CI	
**Innovation**	**0.98**	0	0.98	0.98	**0.98**	0	0.97	0.98	**0.98**	0	0.98	0.98	**1.05**	0	1.04	1.05
**Age at diagnosis**	**1.03**	0	1.03	1.04	**1.03**	0	1.03	1.03	**1.03**	0	1.03	1.04	**0.94**	0	0.94	0.94
CCI*	**1.12**	0	1.11	1.12	**1.13**	0	1.12	1.14	**1.12**	0	1.11	1.12	**0.81**	0	0.80	0.82
**Gender: (ref: Female)**	** **				** **				** **							
**Male**	**1.19**	0	1.18	1.20	**1.25**	0	1.22	1.27	**1.19**	0	1.18	1.20	**0.72**	0	0.70	0.73
**Race (ref: White)**	** **				** **				** **							
**Asian**	**0.82**	0	0.81	0.84	**0.84**	0	0.80	0.87	**0.82**	0	0.81	0.84	**1.41**	0	1.34	1.49
**Other**	**0.93**	0	0.90	0.97	**0.87**	0	0.81	0.94	**0.93**	0	0.90	0.97	**1.14**	0	1.07	1.22
**Black**	**1.05**	0	1.03	1.07	**0.90**	0	0.87	0.94	**1.05**	0	1.03	1.07	**0.93**	0	0.90	0.97
**State of residence (ref: California)**	** **				** **				** **							
**Connecticut**	**0.92**	0	0.90	0.95	**0.99**	0.53	0.95	1.03	**0.92**	0	0.90	0.95	**1.12**	0	1.07	1.17
**Georgia**	**1.00**	0.78	0.98	1.03	**1.14**	0	1.10	1.18	**0.99**	0.45	0.97	1.01	**0.96**	0.083	0.93	1.00
**Hawaii**	**1.08**	0	1.03	1.13	**0.96**	0.34	0.89	1.04	**1.11**	0	1.06	1.16	**0.88**	0.003	0.81	0.96
**Iowa**	**1.01**	0.52	0.98	1.04	**1.10**	0	1.06	1.14	**0.99**	0.62	0.97	1.02	**0.96**	0.146	0.92	1.01
**Michigan**	**0.95**	0	0.94	0.97	**1.05**	0	1.02	1.08	**0.93**	0	0.91	0.94	**1.04**	0.05	1.00	1.09
**New Mexico**	**1.07**	0	1.04	1.11	**1.06**	0.07	0.99	1.13	**1.06**	0	1.02	1.10	**0.84**	0	0.77	0.91
**Utah**	**1.06**	0.02	1.01	1.11	**1.06**	0.14	0.98	1.15	**1.03**	0.21	0.98	1.08	**0.90**	0.039	0.81	0.99
**Washington**	**0.92**	0	0.90	0.94	**1.01**	0.78	0.96	1.05	**0.92**	0	0.89	0.94	**1.02**	0.449	0.97	1.07
Kentucky**	**1.08**	0	1.06	1.10	**1.18**	0	1.13	1.23	**1.05**	0	1.03	1.08	**0.82**	0	0.78	0.86
Louisiana**	**1.09**	0	1.06	1.11	**1.19**	0	1.14	1.24	**1.08**	0	1.05	1.10	**0.81**	0	0.77	0.85
New Jersey**	**0.95**	0	0.93	0.97	**0.99**	0.52	0.95	1.02	**0.95**	0	0.92	0.97	**1.09**	0	1.05	1.14
**Histology (ref: Adenocarcinoma)**	** **				** **				** **							
**Large cell carcinoma**	**1.19**	0	1.16	1.22	**1.21**	0	1.15	1.26	**1.19**	0	1.16	1.23	**0.68**	0	0.64	0.72
**Malignant carcinoma (NOS)**	**1.24**	0	1.21	1.26	**1.22**	0	1.18	1.27	**1.24**	0	1.21	1.27	**0.64**	0	0.61	0.67
**NSCLC**	**1.12**	0	1.11	1.14	**1.15**	0	1.12	1.18	**1.12**	0	1.11	1.14	**0.76**	0	0.73	0.78
**Other**	**0.92**	0	0.90	0.94	**0.89**	0	0.87	0.92	**0.92**	0	0.90	0.94	**1.14**	0	1.10	1.19
**Squamous**	**0.99**	0.54	0.98	1.01	**1.04**	0	1.02	1.07	**0.99**	0.54	0.98	1.01	**0.89**	0	0.86	0.91
**AJCC Stage (ref: IIIA)**	** **				** **				** **							
**IIIB**	**1.72**	0.00	1.67	1.76	**1.59**	0	1.53	1.66	**1.72**	0	1.67	1.76	**0.55**	0	0.53	0.56
**IV**	**2.85**	0.00	2.78	2.92	**2.62**	0	2.53	2.72	**2.85**	0	2.78	2.92	**0.23**	0	0.22	0.24

* R-PFM = Royston-Parmar flexible model; MMV = Mean Medication Vintage; HR = Hazard Ratio, CI = Confidence Interval, CCI = Charlson Comorbidity Index; NOS = Not Otherwise Specified; AJCC = American Joint Committee on Cancer

The results of the regression analyses also demonstrate that older age, more advanced disease at diagnosis, male gender, and higher comorbidity burden were significantly associated with a worse prognosis. As for histology, patients diagnosed with adenocarcinoma had better prognosis than those with large-cell carcinoma. No statistically significant differences were found in the survival of adenocarcinoma patients compared with patients diagnosed with squamous-cell carcinoma; although results of the patient-level exposure (MMV_patient_) analysis did support a negative impact of squamous cell histology on survival. Finally, patients with histology classified as NSCLC or malignant carcinoma had poorer survival.

**Fig 3:**
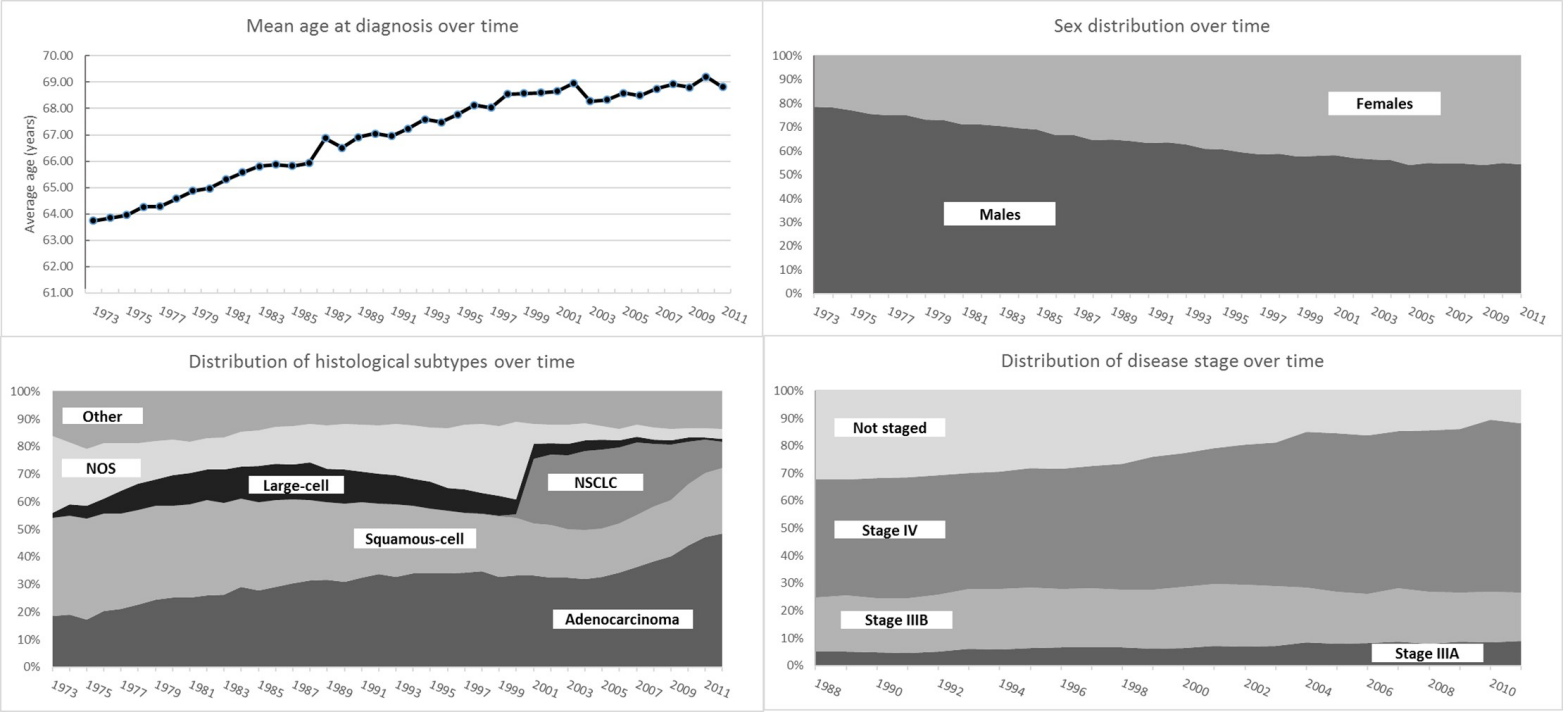
Evolution of patient characteristics over time.

To provide context for the interpretation of these findings, in Fig 3 shows the historical trajectory of the main co-variates. During the study period, the mean age of patients increased as did the percentage of women. Progressively more patients presented with metastatic disease (Stage IV) and fewer were not staged. The histology composition of the cohorts under study in the US varied significantly. At the beginning of the period, squamous-cell carcinoma was the most frequent but as of the early 1990s, more patients were diagnosed with adenocarcinoma. We can also see a shift of Not Otherwise Specified (reduction) and NSCLC (increase) around 2001, indicating a change in classification.

## Discussion

In our study, we established that US patients diagnosed with aNSCLC presented with poor prognosis (9-month median OS), and, despite improvements in one-year OS survival from 14% in 1973, only 39% of patients would survive longer than one year by 2012. During the 40 years between 1973 and 2012, the FDA approved only 10 new systemic therapies for NSCLC with minimal differentiation across subpopulations. The urgent attempts of treating physicians to offer alternatives to their patients is reflected by the off-label use of 28 agents. The reasons for use without an official indication depicts the severity of aNSCLC and the limited treatment options within this disease.

Finding effective treatments for these patients proved particularly arduous due to the heterogeneous nature of NSCLC from a clinical, histological, molecular and biological standpoint [17] as well as the extremely high somatic mutation frequency [18]. A brief review in the Registry and Results Database ClinicalTrials.gov revealed a large number of, thus far, unsuccessful clinical-development programs in aNSCLC. Until recently, much of the efforts devoted to innovate in this indication resulted fruitless. Yet, the shift in focus in aNSCLC clinical development that followed including the creation of a comprehensive catalogue of the somatic mutations responsible for initiation and progression of lung cancer [19], paired with the emergence of immunooncology therapies may be turning the tide. During the past 10 years, we have gained transformative insights into the molecular pathways that play a determinant role in tumor cell growth and proliferation in NSCLC [20], and the consequential advances in clinical and translational research resulted in the approval of 15 new innovative therapies by FDA since 2012 alone. These are dramatically transforming the management of NSCLC. While some new therapies aim to treat the overall aNSCLC indication (like ramucirumab), most of them target subpopulations pre-defined by histology (like necitumumab for squamous-cell and pemetrexed for non-squamous types) or specific markers with the potential to enhance efficacy [20]. The most commonly tested and established biomarkers (and respective therapies/inhibitors) in this indication include Epidermal Growth Factor Receptor (afatinib, gefitinib, and osimertinib), Anaplastic Lymphoma Kinase (alectinib, brigatinib, ceritinib, and crizotinib) and Programmed Death-Ligand 1 (atezolizumab, nivolumab, and pembrolizumab). New targets continue to emerge, such as BRAF mutations (dabrafenib and trametinib) or the ROS1-gene targeted by crizotinib. Numerous meta-analyses have investigated the expected benefits for patients managed with these treatments showing significant gains in progression-free survival [21–27] and impact on OS being currently investigated.

For our study, the 4-month gain in median survival delivered by the 10 new therapies over four decades (and 28 more used off-label) seems low when compared with the threshold of at least a 3.25- to 4-month gain as a measure of meaningful improvement of a new therapy over standard of care recommended by the American Society of Clinical Oncology (ASCO) [28]. The effect of innovation on the expected survival of patients with aNSCLC, during the study period, was also modest (adjusted HR = 0.98), if compared with the target HR for a new therapy recommended by ASCO (HR between 0.76 and 0.8 for squamous and non-squamous respectively) [28]. Although our study showed a high correlation between InnovInd and OS, the small improvement observed in OS, in comparison with ASCO recommendations, may have been impacted by the lower baseline OS at the beginning of our study (14% one-year OS in 1973).

We validated these results through the analysis of Swedish data, specific to the survival outcomes as well as the evolution of patients’ characteristics over time. The proportion of females diagnosed with aNSCLC significantly increased in both countries, which may be the result of gender changes in smoking habits. Prevalence of smoking among women grew steadily following World War II, and continued to increase even while the trend among men has been declining since the 1970’s [7, 29]. Similarly, the proportion of patients diagnosed with different histological types has changed over time with fewer cases of squamous-cell carcinoma, potentially reflecting the shift to low tar and nicotine cigarettes [30]. One of the pitfalls of analyzing aggregate results of patient-level data over a 20-year period may be that the impact of innovation on small subgroups of patients who have achieved greatest benefit could have been diluted in the aggregate analysis. Thus, our aggregated long-term analysis may be masking higher improvement in the 1-year OS of patients diagnosed with adenocarcinoma as compared to those with squamous-cell carcinoma, as demonstrated by Olszewski et al. [31].

An important strength of our study is the use of data from national population-based registries in both countries, which grants these analyses external validity and provides the framework for a natural experiment given the differences in healthcare systems and settings along the entire continuum of care between the US and Sweden and across smaller administrative units (states in the US). Additionally, in this study, we provide a thorough accounting for actual uptake of innovative medicines, even those initially indicated for different cancers and used off-label.

However, a few limitations grant the careful interpretation of the study results. When we evaluated the association between new systemic anti-cancer treatments and outcomes, we controlled for as many potential confounders as possible (age at diagnosis, comorbidities, sex, and disease severity), but data on other important prognostic variables such as performance status were not available. Furthermore, other unaccounted factors such as advances in screening, the precision of diagnostics to detect distant metastases and the overall organization of the treatment continuum may also have influenced the results. Lastly, in the US roughly 60% of the patients have Medicare Part D coverage, including prescription of drugs, and only these drugs are found in SEER-Medicare. Thus, it is possible that we have underestimated the number of treatments, though it is difficult to predict how a shift in the treatment mix may have influenced the analyses. Finally, lack of complete patient-level treatment data, which if consistently collected across the whole study period, would have enabled us to construct a stronger proxy variable to define innovation.

Our analysis of the US SEER Medicare and Swedish cohorts shows that the outlook for aNSCLC patients by the end of 2012 was not optimistic. Yet, the pace at which innovation is being introduced in this indication is accelerating as reflected by the fact that FDA approved ten new chemotherapies in the 40 years before 2012, and 15 new oncology treatments in the five years that followed. Furthermore, the promising initial results of innovative immunotherapies and novel targeted agents suggests that we may be on the brink of a shift in that trajectory and a long-awaited transformational impact on the survival of aNSCLC patients.
